# Acceptance and Factors Influencing Acceptance of COVID-19 Vaccine in a Romanian Population

**DOI:** 10.3390/jpm12030452

**Published:** 2022-03-13

**Authors:** Tiberiu Constantin Ionescu, Bogdana Ioana Fetecau, Ana Giurgiuca, Catalina Tudose

**Affiliations:** 1 Department of Clinical Neurosciences, Faculty of Medicine, University of Medicine and Pharmacy “Carol Davila”, 020021 Bucharest, Romania; tiberiu.ionescu@drd.umfcd.ro (T.C.I.); catalina.tudose52@gmail.com (C.T.); 2 “Prof. Dr. Alexandru Obregia” Clinical Hospital of Psychiatry, 041914 Bucharest, Romania; 3 Department of Cardio-Thoracic Pathology, Faculty of Medicine, University of Medicine and Pharmacy “Carol Davila”, 020021 Bucharest, Romania; bogdana.fetecau@gmail.com

**Keywords:** COVID-19, vaccine, acceptance, refusal, anxiety

## Abstract

COVID-19 vaccination has been recognized as one of the most effective ways to overcome the current SARS-CoV-2 pandemic. However, the success of this effort relies on national vaccination programmes. In May 2021, we surveyed 1552 people from Romania to determine acceptance rates and factors influencing acceptance of a COVID-19 vaccine. Of these, 39.2% of participants reported that they were vaccinated and 25.6% desired vaccination; nonetheless, 29.5% expressed opposition to vaccination. Concerning vaccination refusal, the top justification given by respondents is that the vaccine is insufficiently safe and there is a risk of serious side effects (84.4%). A higher rate of vaccination refusal was observed among female gender, younger age, and lower educational level. Refusal was also associated with unemployment, being in a relationship, and having a decrease in income during the pandemic. People who are constantly informed by specialized medical staff have a statistically significant higher vaccination rate, while people who choose to get information from friends, family, and co-workers have the strongest intention of avoiding the vaccine. Current levels of vaccine are insufficient to achieve herd immunity of 67%. It is mandatory to understand the aspects that define and establish confidence and to craft nationwide interventions appropriately.

## 1. Introduction

In early 2020 the World Health Organization (WHO) announced the novel coronavirus disease 2019 (COVID-19) as a worldwide public health crisis, and in March 2020 the COVID-19 pandemic was declared [1,2]. While governments all over the world instituted protective measures in order to limit infection with the Severe Acute Respiratory Syndrome Corona-2 virus (SARS-CoV-2), the pressure on individuals, health, economic, and social institutions was unprecedented [3].

COVID-19 vaccination has been recognized as one of the most effective ways to address the challenge of this public health crisis [4]. However, according to the most recent estimates, herd immunity for COVID-19 would require at least 67% immunity in the population so that even those not inoculated are protected from COVID-19 [5,6,7]. During the beginning of the COVID-19 vaccination process in Romania, vaccine hesitancy and vaccine-related disinformation spread [8]. Based on numerous surveys, the public’s intention to be vaccinated against COVID-19 was quite modest [9,10,11,12,13]. The scientific and medical community have committed themselves to vaccine research at record speed and strongly encouraged the immunization effort, while a substantial number of laypeople have voiced doubts, uncertainty, and even opposition against COVID-19 vaccinations [9,14,15]. A Twitter poll showed that more than half of the respondents were sceptical about COVID-19 vaccinations [16]. Another study, that examined online search behaviour related to COVID-19 vaccines during the beginning of public vaccination, found that many individuals who are uncertain about critical vaccine-related information do not engage in active online search to address their information needs, highlighting the importance of encouraging more active information-seeking, as well as a critical appraisal of health information on the web, as a strategy for combating misinformation about COVID-19 vaccines [17,18].

**Figure 1:**
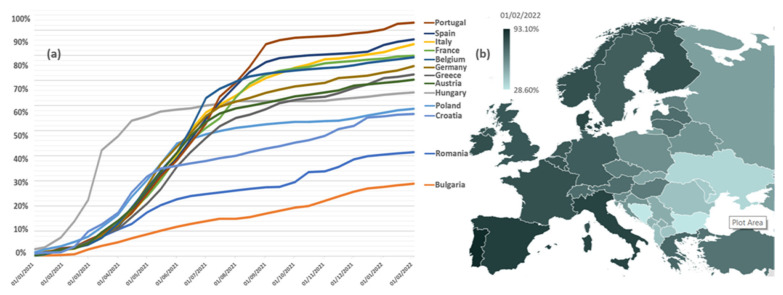
Vaccination rate for COVID-19; (**a**): % of the population having received at least one dose of vaccine, selected countries from Europe, as of 1 February 2022; (**b**): European map of the proportions of people who had received at least one dose of COVID-19 vaccine by country (% of Europe Population, 74.70%; % of Romanian Population, 41.41%) as of 1 February 2022. Data from Johns Hopkins University.

Romania has one of the lowest vaccinations rates among the European countries (Figure 1) [19]. Furthermore, Romania’s healthcare system has the lowest gross domestic product (GDP) expenditure on healthcare and the largest proportion of deaths from curable causes in the European Union (EU) [20]. Consequently, the impact of chronic underfunding of Romania’s public health system immediately became apparent during the pandemic’s early stages [21]. These included, but were not limited to, equipment deficiencies, understaffing of healthcare facilities, insufficient capacity for contact tracing and isolation/quarantine, and general public scepticism about the authorities’ efforts to satisfy population demands [22]. Indeed, significant obstacles were anticipated in Romania with regards to the deployment, distribution, and administration of COVID-19 vaccinations [8,22]. However, the Romanian COVID-19 immunization campaign began at the very same time as the other vaccination efforts around Europe, but vaccine scepticism has emerged as a significant barrier to effectively ending the pandemic [23]. In September 2021, a devastating fourth wave of the COVID-19 pandemic started in Romania, raising worry owing to a quick increase in the number of illnesses and the significant mortality connected with it [24]. By mid-October 2021, Romania, a nation of almost 20 million residents, had reported nearly to 20,000 daily COVID-19 infections and more than 500 everyday deaths, the country’s highest daily mortality rates due to COVID-19 at the time [19].

The current study aims to determine whether there is any evidence of a correlation between low levels of vaccination rates and vaccine acceptance or refusal of vaccination and various socio-demographic features, as well as individual attributes concerning attitudes, behaviours, and anxiety levels.

## 2. Materials and Methods

### 2.1. Participants and Procedures

The present research is a cross-sectional analysis that followed convenience sampling methods. The data were collected between 10 to 31 May 2021, and we used a snowball sampling strategy. The online questionnaire was promoted using social media and other communication tools (the survey was administered using the Survio s.r.o.^®^ (Brno, JM, Czech Republic). The questionnaire was also distributed with help from a non-governmental organization that has access to approximately 150,000 people through Romania’s major social media platforms, and the questionnaire was promoted to this audience. The primary source of income for the organization is the promotion of various products or services to this sample. This sample is made up of people of varying ages, occupations, and social stratification; they have no direct connection to the organization’s main theme. This was done to ensure an objective large visibility of the questionnaire among individuals residing in Romania, and internet users, while minimizing possible collection errors from promoting the questionnaire solely to specific social or professional groups.

### 2.2. Survey Instrument

*Socio-demographic* information was collected on participant gender (male and female), age, residency (urban, peri-urban and rural), marital status (single, married, in relationship, widow, divorced), household size and composition (living alone, living with partner/children/ parents/ siblings/others), number of children (0, 1, 2, ≥3), educational level (secondary school, high school, bachelor’s degree, master’s degree, doctoral degree), mother’s/father’s educational level (no formal education, secondary school, high school, university degree, don’t know), employment status (unemployed, student, employed, freelance, retired), the impact of the COVID-19 pandemic on revenues (significantly decreased, decreased, stagnated, increased, significantly increased) and the religious self-perception (unreligious, religious). Furthermore, the respondents were asked to describe their family background (wealthy—within the highest 25% in Romania in terms of wealth; quite well off—within the 50–75% range for Romania; not very well off—within the 25–50% range for Romania; quite poor—within the lowest 25% in Romania in terms of wealth).

*Medical and psychiatric comorbidities:* Participants were asked if they have any medical and/or psychiatric comorbidities. The response options were binary (yes, no). Those who declared having medical and/or psychiatric conditions to the previous questions were requested to specify the diagnosis. For the regression analysis, the answers were combined as follows: endocrine and metabolic, cardiovascular, rheumatic, and musculoskeletal, neurological, dermatological, gynaecological, pulmonary, other comorbidities (for physical conditions) and major depression disorder, anxiety disorder, and other disorders (for psychiatric conditions), respectively.

*Lifestyle-related characteristics:* The questionnaire also included questions that assessed lifestyle and behaviour characteristics. Weight status included body mass index (BMI) (based on self-reported weight and height), self-assessment on weight (very underweight, slightly underweight, about right, slightly overweight, very overweight), desire to lose weight (yes, no), dieting to lose weight (yes, no). Physical activity over the past 2 weeks (yes, no) and the type of physical activity (walking, fitness, running/ jogging, isometric exercises, cycling, others) were also assessed. Physical activity evaluation in addition included the desire to increased physical activity (yes, no). Finally, the duration of sleep in 24 h period was measured (the answers were combined in 3 categories: <7 h of sleep, 7–8 h of sleep, >8 h of sleep). Satisfaction with their own life (very satisfied, moderately satisfied, no feelings either way, moderately dissatisfied, very dissatisfied) was also evaluated. The measure of lifestyle-related characteristics was adapted from the questions that were used in the International Health and Behaviours Survey (IHBS) [25].

*Anxiety level:* The Self-Rating Anxiety Scale (SAS), developed by Zung, was used to assess anxiety levels. SAS was applied to measure respondents’ anxiety levels. It contains 20 items that cover the most prevalent anxiety symptoms, with a 5/15 ratio of psychological to somatic symptoms, which is why SAS is regarded one of the finest scales for identifying somatic symptoms [26]. Each symptom is rated by the individual on a four-point Likert scale depending on its intensity throughout the previous week.

*COVID-19 pandemic impact:* Furthermore, the questionnaire included a series of questions that assessed the impact of the COVID-19 pandemic on respondents. Participants were asked if they had been infected with SARS-CoV2 (confirmed by a test). Those who were confirmed by a positive test were asked about time elapsed since COVID-19 infection (in months) and their symptomatology (asymptomatic, mild symptoms, moderate symptoms, severe symptoms). Respondents were also asked if someone close to them (a family member or a friend) had been infected or had died from COVID-19. Respondents’ perceptions of the pandemic context and severity of COVID-19 infection were evaluated through single-choice questions with 3 answer options: “SARS-CoV-2 exists and poses a real threat”, “SARS-CoV-2 exists but does not pose a threat”, and “SARS-CoV-2 does not exist”.

Using a 5-point Likert scale, with 1 indicating that they were not influenced at all and 5 indicating that they were completely affected, the survey contained 4 key questions to find out how the pandemic had affected the quality of life of the respondents regarding physical and mental health, work, and social lives. Two multiple-choice questions were used to assess the primary challenges associated with the pandemic (fear of illness, fear of death, fear of illness of close people, fear of death of close people, concerns regarding the capacity of the health system, fear that the basic protective measures like masks or disinfection of hands are not enough, concerns regarding the risk of becoming unemployed/economic impact, social isolation, social stigma associated with COVID-19 infection, family conflicts) and which are their main sources of information regarding SARS-CoV-2 infection (traditional media—television, newspapers, and radio broadcasting; the internet—blogs, news websites, and social media; friends, co-workers, and family members; medical staff). COVID-19 pandemic impact questions were taken from Ionescu et al. [27].

*Results on COVID-19 vaccination acceptance:* Our main outcome measure was vaccine acceptance (got vaccinated; want to get vaccinated; do not want to get vaccinated; contraindication to vaccine—self reported). The main reason for refusing and for contraindication was also evaluated. According to the current study’s design, acceptance of vaccination was determined by the fact that respondents had either been vaccinated or wanted to get vaccinated at the time when the survey was conducted.

### 2.3. Quality Control

**Figure 2:**
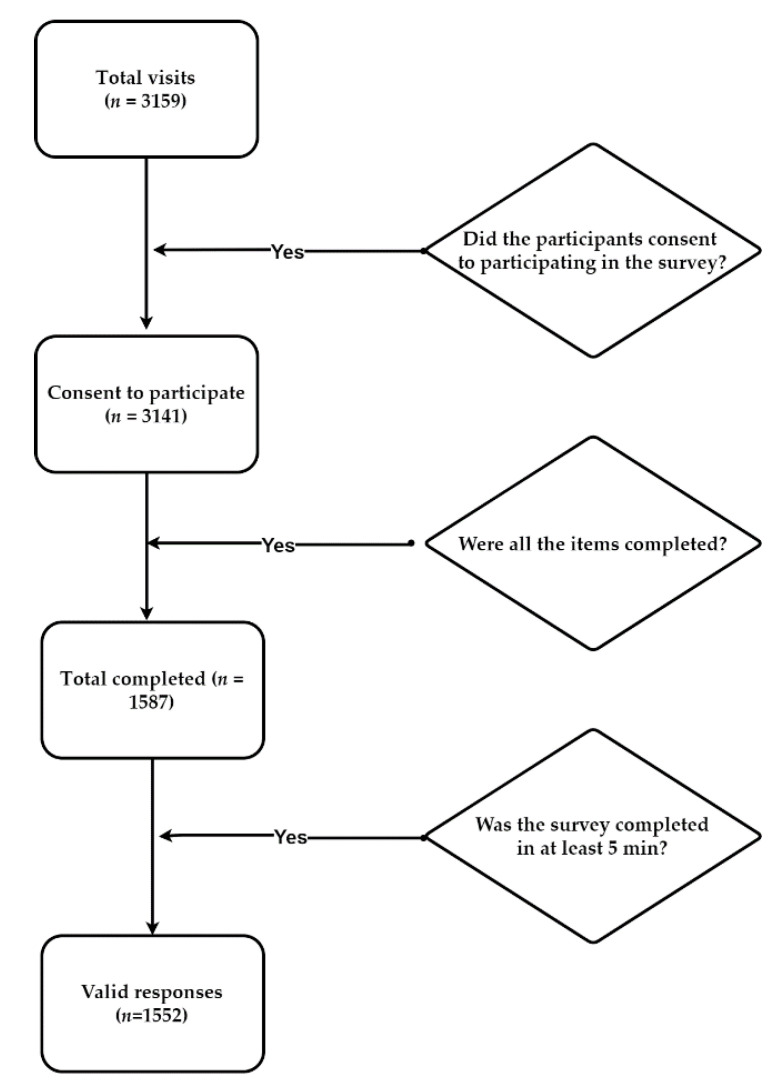
The number of unique visitors was 3159, of which 3141 gave their consent to participate and accessed the first survey page (99.43% participation rate); 1587 out of 3141 users completed the questionnaire (50.52% completion rate); 1552 out of 1587 completed the questionnaire in at least 5 min. The response rate was 49.31% with 1552 responses from 3159 unique visitors.

The same IP address was limited to providing a single response, and all entries were marked as necessary. The questionnaire was sent only once all items were completed. Otherwise, the system flagged the result as incomplete. Pre-test scores were used to determine the test duration, and answers with survey duration of less than 5 min were removed. Subjects were informed of the purpose and significance of this study before online psychological assessments and all participants provided informed consent (Figure 2).

### 2.4. Ethics Considerations

The study was developed in accordance with the World Health Organization (WHO), the Declaration of Helsinki, European Union legislation, and the ethical principles of clinical research of the Guidelines for Good Clinical Practice (ICH-GCP).

The protocol and the informed consent were approved by the Research Ethics Committee of University of Medicine and Pharmacy “Carol Davila” Bucharest (PO-35-F-03/7 May 2021).

### 2.5. Statistical Analyses

IBM^®^ SPSS^®^ Statistics version 26 was used to analyse the data (IBM^®^, Armonk, NY, USA). Along with descriptive statistics, we did univariate analyses to determine the connections between anxiety symptoms and associated factors using the Student’s *t*-test, ANOVA test or the Pearson’s correlation test. Then, using multiple linear regression analyses, we determined the unique effect of relevant predictors on the SAS total and subscale scores, respectively. All analyses were two-tailed with alpha set at 0.05.

## 3. Results

### 3.1. Socio-Demographic Characteristics

**Figure A1:**
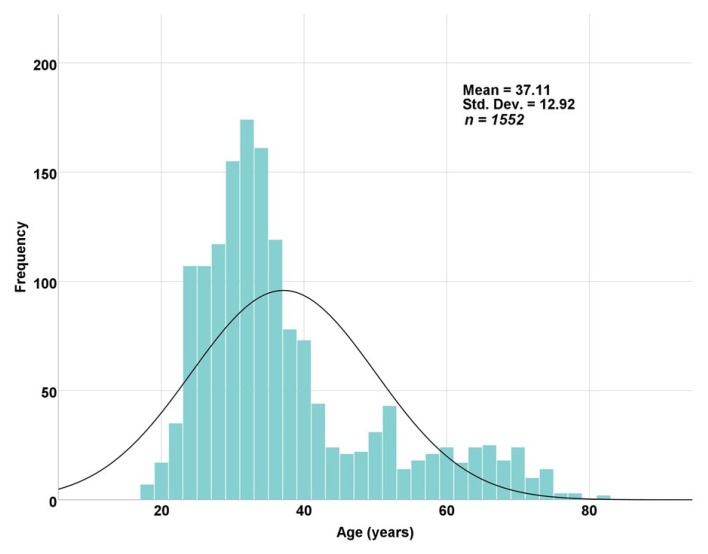
Histogram by age. *n* = 1552; the most representative age group was 25–44 years old (67.8%, *n* = 1052). Abbreviation: Std. Dev., standard deviation.

The final sample consisted of 1552 participants, 78.4% female (*n* = 1217), and the most representative age group was 25–44 years old (Figure A1); 86.2% were married or in a stable relationship and 75.0% living with a partner (Table A1). One in two were employed (49.4%) and for more than a quarter of the subjects their revenues decreased during the COVID-19 pandemic (26.3%).

In terms of family background, 1.4% (*n* = 22) described their own family as wealthy, while 46.8% (*n* = 726) described their own family as quite well off. On the other hand, 49.8% (*n* = 773) considered themselves to be not very well off and 1.3% (*n* = 30) considered their family to be quite poor.

### 3.2. Medical and Psychiatric Comorbidities

A pre-existing physical condition was found in 29.4% of the sample (*n* = 456), with endocrine and metabolic disorders (7.8%) being the most common, followed closely by cardiovascular disease (7.1%) (Table A2). Rheumatic and musculoskeletal problems (3%), as well as hepatic diseases (2.7%), are also among the most prevalent pre-existing pathologies among participants. In terms of psychiatric disorders, 5.6% of participants (*n* = 87) reported having a pre-existing diagnosis, with major depressive disorder and anxiety disorders being the most reported. Independent of pre-existing physical or mental comorbidities, 21.1% (*n* = 327) of the respondents had experienced health difficulties that necessitated a visit to the doctor or a health clinic over the last four weeks, according to the survey. Furthermore, 55.6% (*n* = 863) of them had taken medication that was prescribed by a doctor (25.8%, *n* = 400) or purchased directly from a drugstore (29.8%, *n* = 463) in the last four weeks.

### 3.3. Lifestyle-Related Characteristics

In terms of weight status, more than half of the participants (52.8%, *n* = 819) had a BMI that is classified as normal weight (Table A3). Most respondents (58.6%, *n* = 909) reported engaging in physical activity in the previous two weeks. Walking (14.0%, *n* = 218), fitness (13.1%, *n* = 204), running (10.4%, *n* = 161), and isometric exercises (7.9%, *n* = 123) were among the most frequently reported physical activities. In terms of average sleep duration, most respondents (64.6%, *n* = 1003) stated that they slept 7–8 h per day. 

Overall, 83.2% (*n* = 1290) of participants were satisfied with their own lives (moderately satisfied, 60.4% and extremely satisfied, 22.8%), while 3.9% (*n* = 60) were dissatisfied (moderately dissatisfied, 3.2% and extremely dissatisfied, 0.7%). A total of 201 participants (13.0%) said they were neither satisfied nor dissatisfied with their personal lives.

### 3.4. Anxiety Levels

**Figure 3:**
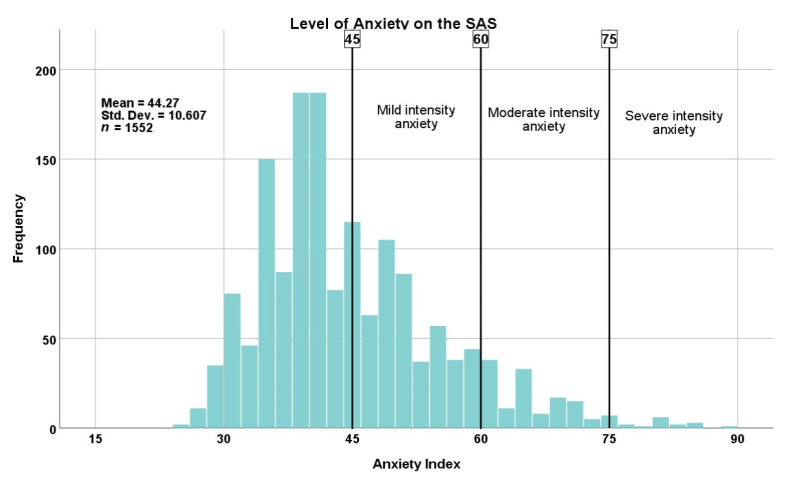
Level of anxiety on the SAS (Self-Rating Anxiety Scale). In total, 41.3% of the respondents (*n* = 641) reported high levels of anxiety: mild intensity 31.7% (*n* = 492), moderate intensity 8.4% (*n* = 130), severe intensity 1.2% (*n* = 19). Abbreviations: Std. Dev., standard deviation.

Concerning anxiety level, 4 out of 10 respondents (41.3%, *n* = 641) reported anxiety symptoms (Figure 3). Furthermore, 8.4% reported moderate-intensity symptoms and 1.2% reported severe-intensity symptoms. The mean anxiety index and standard deviation (SD) were 44.27 and 10.60, respectively. Only 10.3% (*n* = 66) of respondents who scored above the threshold value on the SAS were diagnosed with a psychiatric disorder, with major depression (5.1%, *n* = 33) and anxiety disorders (3.9%, *n* = 25) being the most frequently diagnosed.

In particular, the female participants scored significantly higher compared to males (mean female anxiety index = 45.21, mean male anxiety index = 40.89, *p* < 0.001) and respondents under 25 years old were more likely to experience symptoms of anxiety (mean anxiety index = 48.33, SD = 12.49) (*p* < 0.001) (Table A4).

**Figure 4:**
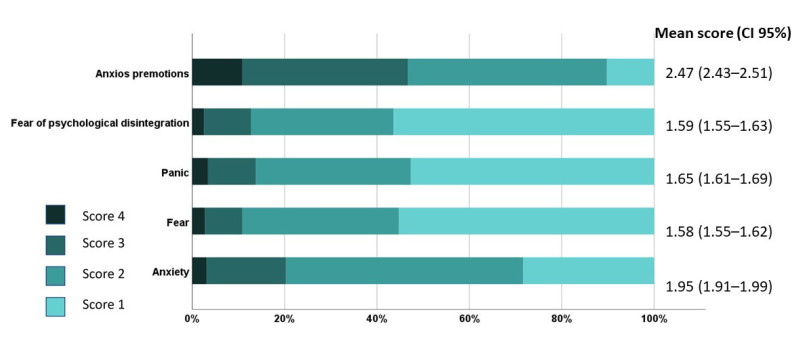
Average scores for each psychological item of the Zung Self-Rating Anxiety Scale (SAS) in all participants (*n* = 1552). The highest score in a single item of the SAS is 4 (i.e., most of the time/always/almost always) and the lowest score is 1 (i.e., a little of the time/very rarely/rarely). Abbreviation: CI, confidence interval.

In the psychological subdomain of SAS, the question evaluating apprehensive premonitions received the highest score out of the five questions evaluating psychological symptoms of anxiety (Figure 4). Psychological symptoms were statistically more frequently related to female gender (*p* < 0.001), younger age (*p* < 0.001), unemployed and retired participants (*p* = 0.030), number of children (*p* = 0.048), and income decline during the pandemic (*p* < 0.001).

**Figure 5:**
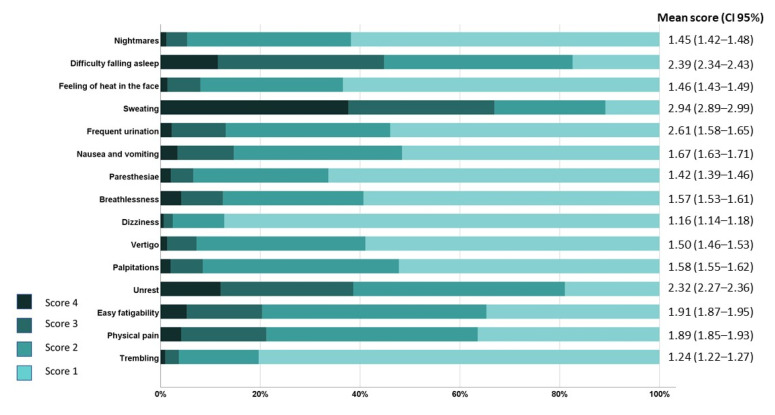
Average scores for each somatic item of the Zung Self-Rating Anxiety Scale (SAS) in all participants (*n* = 1552). The highest score in a single item of the SAS is 4 (i.e., most of the time/always/almost always) and the lowest score is 1 (i.e., a little of the time/very rarely/rarely).

Regarding the somatic subdomain of SAS, out of the 15 items which evaluated the physical/somatic manifestations of anxiety, the three most common symptoms were sweating, unrest, and difficulty in falling asleep (Figure 5). Similar to psychological symptoms, somatic symptoms were also statistically more frequently related to female gender (*p* < 0.001), younger age (*p* < 0.001), number of children (*p* = 0.048), and income decline during the pandemic (*p* < 0.001).

### 3.5. COVID-19 Pandemic Impact 

**Figure 6:**
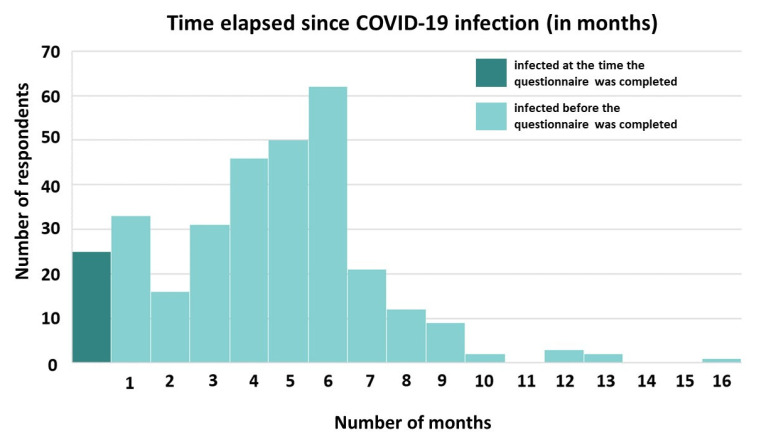
Distribution of the length of time (in months) elapsed since respondents were confirmed with SARS-CoV-2 infection. In total, 263 of respondents were infected in the last 6 months.

In total, 20.2% (*n* = 313) of the participants were confirmed to have had a SARS-CoV-2 infection, of whom 8.0% (*n* = 25) were infected at the time of completing the questionnaire, while the rest were diagnosed with COVID-19 between one and sixteen months before (Figure 6).

In terms of COVID-19 illness severity, 7.0% (*n* = 22) of the participants were asymptomatic, 48.6% (*n* = 152) had mild symptoms, 38.0% (*n* = 119) had moderate symptoms, and 6.4% (*n* = 20) showed severe symptoms. Most of the respondents (94.2%, *n* = 295) who were infected with SARS-CoV-2 had isolated at home, while just 5.8% (*n* = 18) were hospitalized. The most frequently reported symptoms of infection were fatigue (69.0%, *n* = 216), loss of smell (65.8%, *n* = 206), loss of taste (57.5%, *n* = 180), headaches (54.9%, *n* = 172), muscle aches (49.2%, *n* = 154), sore throats (32.2%, *n* = 101), fever (33.2%, *n* = 104), cough (25.8%, *n* = 81), and dyspnoea (20.1%, *n* = 63).

Concerning the status of COVID-19 among those in their immediate social environment (family or friends), 74.5% (*n* = 1156) reported knowing someone who had been infected, and 15.4% (*n* = 239) reported knowing someone who died of COVID-19.

**Figure 7:**
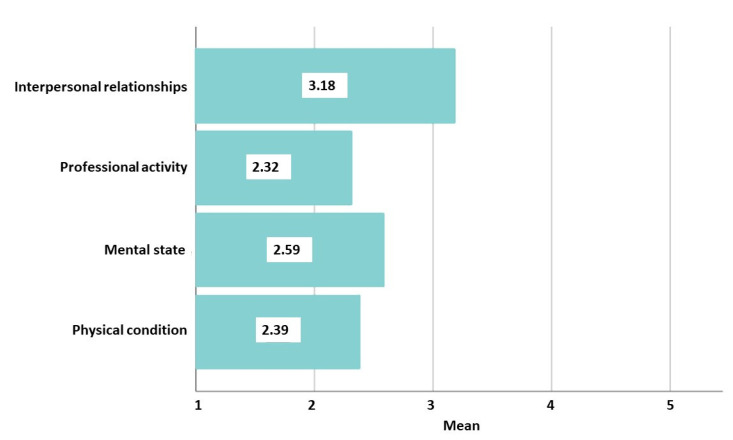
The impact of the pandemic on functionality. Using 5-point Likert scales (1 means not at all, and 5 means completely affected), functioning was evaluated in four areas. Interpersonal relationships (mean = 3.18, standard deviation (SD) = 1.23) and mental state (mean = 2.59, SD = 1.16) were the most affected by the pandemic, followed by impaired physical condition (mean = 2.39, SD = 1.10), and professional/academic activities (mean = 2.32, SD = 1.30).

According to respondents’ perceptions of the pandemic context and severity of COVID-19 infection, the majority (71.1%, *n* = 1103) believe that SARS-CoV-2 exists and poses a real threat, while more than a quarter believe the virus exists but does not pose a threat (27.1%, *n* = 420), or the virus does not exist (1.9%, *n* = 29). The impact of the pandemic on functionality was felt most strongly in the impact on personal relationships. (Figure 7).

Main concerns associated with the pandemic context include fear of illness and death of close relatives (63.1%, *n* = 980, and 48.1%, *n* = 747, respectively), concerns about the health system’s capacity (53.9%, *n* = 836), social isolation (44.4%, *n* = 689), fear of illness (33.6%, *n* = 522), and concerns about unemployment or economic impact (21.3%, *n* = 330). (Table 1).

The internet (blogs, news websites, and social media) and medical staff in healthcare settings are the primary sources of information about the pandemic and primary prevention measures, resulting in an equal number of responses (59.5% for each source of information), followed by traditional media such as television, newspapers, and radio broadcasting (48.4%), and, to a lesser extent, friends, co-workers, and family members (30.9%) (Table 2).

### 3.6. Results on COVID-19 Vaccination Acceptance 

At the time of the study, 39.2% of participants were vaccinated and 25.6% desired vaccination; 29.5% expressed opposition to vaccination. Concerning vaccination refusal, the primary argument mentioned by respondents was that the vaccine is insufficiently safe and there is a possibility of major side effects (Table 3).

A lower vaccination rate, as well as a greater degree of refusal to receive COVID-19 vaccination, was associated with female gender, a lower educational level, unemployment status, living alone, not being a parent, and a significantly decreased income during the pandemic (Table 4).

Vaccination intention is directly proportional to economic status, so respondents who consider their family wealthy had the highest vaccination rate (*p* < 0.001).

Individuals with documented somatic comorbidities, particularly cardiovascular and metabolic comorbidities, and those who had been exposed to SARS-CoV-2 from their proximal group of people (friends and family), were also associated with a high rate of COVID-19 vaccination.

In terms of lifestyle and varied behaviours, a higher vaccination rate was reported among those who wished to lose weight and attempt dieting (*p* = 0.002), as well as those who wished to increase their physical activity (*p* < 0.001) (Table 5). According to BMI and self-assessment, being overweight was associated with a statistically significant increased vaccination rate. The intention to vaccinate was also significantly associated with 24 h average sleep duration.

**Figure 8:**
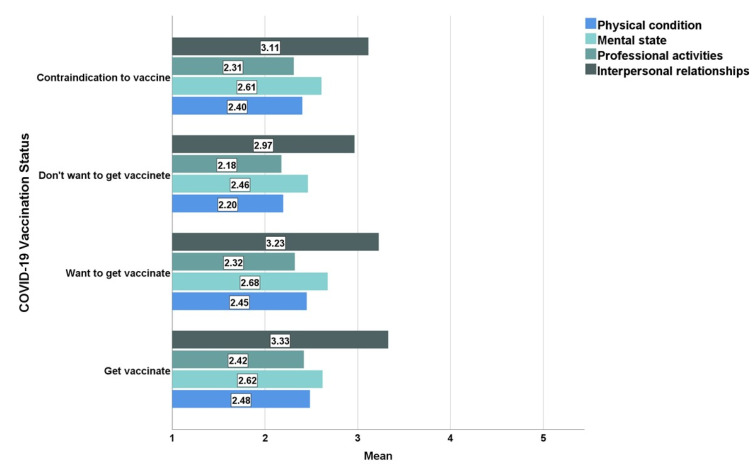
The impact of the pandemic on functionality and COVID-19 vaccination status.

Furthermore, those who perceived the pandemic as having a severe effect on their physical condition (*p* = 0.001), professional activities (*p* = 0.010), and interpersonal relationships (*p* < 0.001) indicated a greater vaccination rate and intention to vaccinate, in contrast to those who believed the pandemic affected their mental state, which was not statistically associated with a higher vaccination rate or intention to vaccinate (*p* = 0.093) (Figure 8).

As expected, the highest vaccination rate was recorded among people who believe that the SARS-CoV-2 virus really exists and is a real threat (48.78% of this sample were vaccinated, 29.47% wanted to be vaccinated at the time of the study, while only 16.59% refused vaccination), unlike those who believe that the virus exists but does not pose a threat (16.67% were vaccinated, 16.90% wanted to be vaccinated, and 59.52% refused vaccination) and those who considered that SARS-CoV-2 did not exist (3.45% were vaccinated, 6.90% wanted to be vaccinated, and 86.21% refused vaccination) (Pearson’s *r* = 0.381, *p* < 0.001).

Moreover, a higher vaccination rate and a higher desire for vaccination were registered among those who were afraid of infection (43.44% were vaccinated, 31.08% want to be vaccinated, Pearson’s *r* = −0.100, *p* < 0.001) and death (42.80% were vaccinated, 30.63% want to be vaccinated, Pearson’s *r* = −0.050, *p* = 0.05), and of those who fear that close people could be infected (43.90% were vaccinated, 28.62% want to get vaccinated, Pearson’s *r* = −0.170, *p* < 0.001), or may die due to COVID-19 (44.95% have been vaccinated, 30.28% want to get vaccinated, Pearson’s *r* = −0.169, *p* < 0.001) (Table 6). Furthermore, a higher vaccination rate and a higher vaccination intention were also registered among those who consider that the medical system is over capacitated.

On the other hand, those who associated the pandemic with the fear of losing their job and financial stability, as well as those who were worried about possible conflicts within the family, statistically showed a greater refusal rate to vaccination (Pearson’s *r* = 0.81, *p* = 0.001, Pearson’s *r* = 0.55, *p* = 0.031, respectively).

Regarding the relationship between COVID-19 vaccination status and the primary sources of information on SARS-CoV-2 infection, it was observed that people who were constantly informed by specialized medical staff had a statistically significantly higher vaccination rate, whereas people who chose to get information from friends, family, and co-workers had the strongest intention of refusing the vaccine (Table 7).

An association between anxiety level on SAS and anti-COVID-19 vaccination status, as well as between SAS psychological and somatic subdomains, could not be established. The level of anxiety, on the other hand, was statistically linked to a variety of current-context attitudes and behaviours, which in turn affects how responders interpreted the COVID-19 immunization campaign. Statistical correlations were observed between the level of anxiety and how respondents perceived SARS-CoV-2. In comparison to those who believe the virus exists and is a serious threat (mean anxiety index = 43) and those who believe the virus exists but is not a threat (mean anxiety index = 40), those who believe SARS-CoV-2 does not exist had the highest level of worry (mean anxiety index = 46) (*p* = 0.010). Regarding infection with the SARS-CoV-2 virus, a higher level of anxiety was also associated with the severity of symptoms in individuals infected and the necessity of hospital admission.

Participants with symptoms of moderate and severe COVID-19 reported significantly higher levels of anxiety than those who were asymptomatic or only had mild symptoms, according to the results of the study (44.00 and 53.00, respectively, compared to 40.50 and 40.00, respectively, *p* = 0.001), and those who were hospitalized were more anxious than those who were not (mean anxiety index of 45.50 compared to 41.00, *p* = 0.003). Dyspnoea, headaches, and fatigue were found to be associated with higher levels of anxiety in patients with COVID-19 infection (Table 8). Furthermore, a high degree of anxiety was also statistically associated with a sense of decreased physical (*p* < 0.001) and mental status (*p* < 0.001), as well as negative professional impact (*p* < 0.001) and affected interpersonal relationships (*p* < 0.001).

## 4. Discussion

Great measures have been taken in order to overcome the COVID-19 pandemic and the development of specific vaccines came as a hopeful opportunity for achieving herd immunity all over the world. However, reluctancy to get vaccinated has hindered the efforts of the Romanian vaccination programme and created a great burden on the already underfunded and overwhelmed medical system. To prevent the spread of the virus, we postulated that it is critical to identify the reasons why people refuse to be vaccinated in Romania. With a thorough understanding of these factors, vaccination campaigns may be tailored to the specific needs of each community.

According to the current study’s design, acceptance of vaccination was determined by the fact that respondents had either been vaccinated or wanted to get vaccinated at the time when the survey was conducted. A higher rate of vaccination refusal was observed among respondents of the female gender, aged 25–44, and with a lower level of education. Unwillingness to vaccinate was also associated with unemployment, being in a relationship, not being a parent, and having a decrease in income during the pandemic. A recent meta-analysis confirmed a lower rate of vaccination intentions among women, which may be attributed to females’ high levels of mistrust about vaccine benefits and more negative concerns about future unpredicted side effects, both of which are important determinants of the intention to be vaccinated [28,29]. This trend may have been amplified in the current research by the fact that females are more likely to utilize social media and are therefore overrepresented in our sample. Replicating the findings of prior studies, we found that the 25–44 age group was less likely to get vaccinated than both the younger (18–25 years) and older (>45 years) groups [30]. On the other hand, a study conducted in Greece by Mouliou et al. found a high incidence of immunization against COVID-19 among women and young individuals, but a lower rate among those with a lower level of education, as our study found [31]. Our findings have confirmed previous findings suggesting those from lower socioeconomic communities, described as lower family wealth, poorer educational achievement, unemployment, and declining wages due to the pandemic, have been found to have higher rates refusal to accept vaccination. This may be because people in these communities and groups of people do not trust public sector officials or the government [32,33]. Furthermore, people who are single and/or lived alone had much higher vaccination acceptance rates than those who lived with others (partners, family members, friends, or housemates). The current results are in contrast with prior findings that, in both the United States and the United Kingdom, more people would accept a vaccine to protect family, friends, or vulnerable groups than to protect themselves [10]. The current finding supports the notion that older people are more aware of the harmful consequences of COVID-19 and hence more ready to get vaccinated [9,11]. Participants with somatic comorbidities, particularly cardiovascular and metabolic comorbidities, and those who had people infected with SARS-CoV-2 in their close social network (friends, family) had a higher rate of acceptance of vaccination, compared to those without such circumstances (they were or wanted to be vaccinated).

Our findings on the influence of worries and other psychological and behavioural characteristics in predicting the desire to get vaccinated against COVID-19 are essentially comparable with those of earlier studies [34,35,36,37]. People who believe the SARS-CoV-2 virus exists and poses a serious threat had the highest vaccination rate, while those who believe the virus exists but does not pose a threat and those who believe SARS-CoV-2 does not exist had a lower vaccination rate. Those who feared infection and death from COVID-19, as well as those who worried about the infection or death of those close to them, had higher vaccination rates and a greater desire for immunization. In addition, people who believe that the medical system is deficient have a greater desire to vaccinate.

People who were constantly informed by specialized medical staff had a statistically significant higher vaccination rate, while people who chose to get information from friends, family, and co-workers had the strongest unwillingness toward getting vaccinated. Furthermore, previous research has indicated that recommendations from health officials as the first source of information about the COVID-19 pandemic are statistically associated with a high degree of confidence in immunization [32]. Consequently, high levels of vaccination acceptance require trust in science and medical staff. Additionally, numerous studies support the hypothesis according to which COVID-19 immunization is more prevalent among individuals who put a high value on science and follow COVID-19 prevention instructions more carefully [30,38,39,40,41,42,43].

Unlike vaccination rates in Western Europe, the countries of the former communist bloc are facing a high reluctance to vaccinate. According to the results of the survey, 39.2% were vaccinated and 25.6% wanted vaccines; nevertheless, 29.5% indicated hostility towards vaccination. Similar results were recorded in other studies that were conducted in Romania [23,27] and in other Eastern European countries [44,45,46,47]. At the time of performing the survey, between 10 May and 31 May 2021, proof of vaccination was not mandatory in Romania, and it was not prohibited in Romania to visit any public locations without being vaccinated. From 15 May, the Romanian government eased COVID-19-related domestic restrictions and maintained international entry measures, depending on the origin country’s risk level. The European Union Digital COVID Certificate (EUDCC) entered into application on 1 July 2021 [48]. On 31 May 2021, the vaccination rate by at least one dose among the population of Romania was roughly 22.0%, which was lower than the vaccination rate among the respondents examined in this study, which was 39.2% at the time [19]. The reason for this disparity might be attributed to a variety of socio-demographic factors. First and foremost, Romania has a high migration rate; in 2011, it was estimated that more than 3.5 million Romanians had left the country in the last two decades, accounting for more than 17% of the country’s total population [49,50]. This means that the true percentage of vaccinated people in the population of Romania is higher than what is currently estimated by the national statistics. Secondly, 84.7% of respondents lived in urban and peri-urban areas, where vaccination rates are higher than in rural areas. A possible difference in vaccination rate between urban and rural areas can be attributed to both the significantly higher concentration of vaccination centres in urban settlements and the increased reluctance towards vaccinations among people in rural areas [23,51]. Concerning vaccination refusal, the top reason given by respondents is that the vaccine is insufficiently safe and there is a risk of serious side effects (84.4%). Numerous factors contribute to people’s growing reluctance to accept COVID-19 vaccines, including concerns about vaccine effectiveness, safety, and adverse effects; the belief that the vaccine is limited, and other people require it more; a general lack of trust in vaccines; or that the impact of COVID-19 is greatly exaggerated [52,53]. Additionally, other studies conducted in 2021 indicate that fear of short-, medium-, and long-term negative effects is the primary cause for refusal (35–60%) [33,53,54].

The major addition of the current study to the current scientific data is that we provided an updated picture of vaccination trends in 2021, during a period in which the Romanian government had only recently launched its immunization campaign, whereas previous studies were mostly conducted during the first lockdown phase in 2020 [55,56]. Furthermore, it is noteworthy that participants were mostly recruited through social media, given that the spread of disinformation appears to be particularly rapid on social media platforms.

Limitations include the assumption that our sample was not representative of the general Romanian population in the view of the fact that participants were recruited through social media channels using a snowball sampling strategy, and were, as a result, predominately female, mainly aged between 25 to 45 years old, and with a high level of educational achievement. Secondly, because our study was cross-sectional and non-interventional in nature, we could not report on modifying factors concerning attitudes toward vaccination in the Romanian population. Future research should take these elements and more variables into account to gain a better understanding of the complicated process that underlies people’s intention to be vaccinated.

As our data and multiple studies show, in order to achieve herd immunity, it is not enough to ensure that all people have equal access to COVID-19 vaccines, but also that health authorities and governments gain the confidence of their people and develop efficient vaccination programmes to raise acceptance and decrease unwillingness for SARS-CoV-2 immunization. In order to promote trust among the general public, it is necessary to understand the aspects that define and establish confidence and to craft interventions appropriately. It is becoming increasingly obvious that all key stakeholders will be expected to engage in open, evidence-based policymaking and clear, accurate communication. The current pandemic presents a unique opportunity for the public health systems to increase vaccine awareness and confidence to facilitate the adoption of the COVID-19 vaccine and to strengthen the overall vaccination efforts for other future vaccine-preventable illnesses.

## Figures and Tables

**Table 1:** Respondents were asked how concerned they were about different health, social, economic, and family-related situations in the context of COVID-19.

Main Concerns Associated with the Pandemic Context	*n*	Responses (%)	Respondents (%)
Fear of illness	522	10.8%	33.6%
Fear of death	275	5.7%	17.7%
Fear of illness of close people	980	20.3%	63.1%
Fear of death of close people	747	15.5%	48.1%
Concerns regarding the capacity of the health system	836	17.3%	53.9%
Fear that the basic protective measures (mask, disinfection of hands) are not enough	256	5.3%	16.5%
Concerns regarding the risk of becoming unemployed/economic impact	330	6.8%	21.3%
Social isolation	689	14.3%	44.4%
Social stigma associated with COVID-19 infection	156	3.2%	10.1%
Family conflicts	32	0.7%	2.1%
Total	4823	100.0%	

**Table 2:** Variety of sources from which respondents get information on COVID-19.

Sources of Information	*n*	Responses (%)	Respondents (%)
Traditional media (television, newspapers, and radio broadcasting)	751	24.4%	48.4%
The internet (blogs, news websites, and social media)	923	30.0%	59.5%
Friends, co-workers, and family members	479	15.6%	30.9%
Medical staff	924	30.0%	59.5%
Total	3077	100.0%	

**Table 3:** Reasons given by respondents who were unwilling to take COVID-19 vaccine presented as percentages of responses (*n* = 494) and respondents (*n* = 458).

The Main Reason for Refusal Is the Following	Responses (%)	Respondents (%)
I’m not afraid to get infected	7.3%	7.8%
There is not enough evidence of vaccine safety	84.4%	90.5%
I don’t think the COVID-19 disease exists	5.9%	6.3%
Others	2.4%	2.6%
Total	100.0%	107.2%

**Table 4:** Bivariate analysis for association between socio-demographic characteristics and intention to COVID-19 vaccination (*n* = 1552).

Socio-Demographic Characteristics		COVID-19 Vaccination Status % (*n*)				*p* Value
		Got Vaccinated	Want to Get Vaccinated	Don’t Want to Get Vaccinated	Contraindication to Vaccine	
Gender	Female	34.18% (416)	27.36% (333)	32.29% (393)	6.16% (75)	<0.001 **
	Male	57.61% (193)	19.4% (65)	19.4% (65)	3.58% (12)	
Age Groups	<25	49.4% (82)	19.28% (32)	27.11% (45)	4.22% (7)	<0.001 **
	25–34	28.85% (206)	30.53% (218)	33.75% (241)	6.86% (49)	
	35–44	36.98% (125)	28.99% (98)	28.99% (98)	5.03% (17)	
	45–54	47.33% (62)	20.61% (27)	26.72% (35)	5.34% (7)	
	55–64	54.81% (57)	18.27% (19)	23.08% (24)	3.85% (4)	
	>65	77.78% (77)	4.04% (4)	15.15% (15)	3.03% (3)	
Educational level	Secondary school	6.67% (2)	40.00% (12)	53.33% (16)	0.00% (0)	<0.001 **
	Highschool	30.05% (128)	21.13% (90)	43.43% (185)	5.4% (23)	
	University graduate	43.22% (252)	26.07% (152)	25.04% (146)	5.66% (33)	
	Master graduate	41.36% (170)	29.2% (120)	23.11% (95)	6.33% (26)	
	Doctoral degree	57.00% (57)	23.00% (23)	15.00% (15)	5.00% (5)	
Occupational status	Unemployed	18.98% (75)	31.40% (124)	40.76% (161)	8.86% (35)	<0.001 **
	Employed	38.64% (296)	26.89% (206)	29.5% (226)	4.96% (38)	
	Student	70.47% (105)	18.12% (27)	8.05% (12)	3.36% (5)	
	Retired	71.09% (91)	8.59% (11)	16.41% (21)	3.91% (5)	
	Freelancer	37.17% (42)	26.55% (30)	33.63% (38)	2.65% (3)	
Family background	Wealthy	68.18% (15)	22.73% (5)	4.55% (1)	4.55% (1)	<0.001 **
	Quite well off	43.39 (315)	23.14% (168)	27.82% (202)	5.65% (41)	
	Not very well off	35.19% (272)	27.55% (213)	31.57% (244)	5.69% (44)	
	Quite poor	23.33% (7)	36.67% (11)	36.67% (11)	3.33% (1)	
Being single	No	37.65% (532)	25.83% (365)	30.64% (433)	5.87% (83)	<0.001 **
	Yes	55.4% (77)	23.74% (33)	17.99% (25)	2.88% (4)	
Living alone	No	38.4% (520)	25.33% (343)	30.35% (411)	5.91% (80)	=0.014 *
	Yes	44.95% (89)	27.78% (55)	23.74% (47)	3.54% (7)	
Being parents	No	51.59% (211)	23.96% (98)	20.54% (84)	3.91% (16)	<0.001 **
	Yes	34.82% (398)	26.25% (300)	32.72% (374)	6.21% (71)	
Religious self-perception	Unreligious	48.05% (259)	27.27% (147)	21.15% (114)	3.53% (19)	<0.001 **
	Religious	34.55% (350)	24.78% (251)	33.96% (344)	6.71% (68)	
Physical comorbidities(s)	No	33.76% (370)	28.38% (311)	32.66% (358)	5.2% (57)	<0.001 **
	Yes	52.41% (239)	19.08% (87)	21.93% (100)	6.58% (30)	
Cardiovascular condition	No	37.17% (536)	26.63% (384)	30.44% (439)	5.76% (83)	<0.001 **
	Yes	66.36% (73)	12.73% (14)	17.27% (19)	3.64% (4)	
Metabolic condition	No	38.11% (574)	26.29% (396)	29.95% (451)	5.64% (85)	<0.001 **
	Yes	76.09% (35)	4.35% (2)	15.22% (7)	4.35% (2)	
Revenues (compared to pre pandemic period)	Significantly decreased	18.39% (16)	35.63% (31)	39.08% (34)	6.9% (6)	<0.001 **
	Decreased	32.92% (106)	26.4% (85)	34.47% (111)	6.21% (20)	
	Stagnated	41.99% (406)	24.61% (238)	27.82% (269)	5.58% (54)	
	Increased	44.37% (67)	25.83% (39)	25.83% (39)	3.97% (6)	
	Significantly Increased	56.00% (14)	20.00% (5)	20.00% (5)	4.00% (1)	
Knowing someone with the COVID-19 infection	No	28.79% (114)	22.22% (88)	40.91% (162)	8.08% (32)	
	Yes	42.82% (495)	26.82% (310)	25.61% (296)	4.76% (55)	<0.001 **

* Correlation is significant at the 0.05 level (2-tailed). ** Correlation is significant at the 0.01 level (2-tailed).

**Table 5:** Bivariate analysis for association between intention to COVID-19 vaccination and participants’ weight, physical activity, and sleep duration (*n* = 1552).

Lifestyle-Related Characteristics		COVID-19 Vaccination Status % (*n*)				*p* Value
		Got Vaccinated	Want to Get Vaccinated	Don’t Want to Get Vaccinated	Contraindication to Vaccine	
BMI (kg/m^2^)	Underweight	26.09% (24)	28.26% (26)	39.13% (36)	6.52% (6)	=0.012 *
	Normal range	37.65% (305)	26.54% (215)	29.88% (242)	5.93% (48)	
	Overweight	42.04% (177)	25.65% (108)	28.50% (120)	3.80% (16)	
	Obese	44.60% (95)	21.60% (46)	26.29% (56)	7.51% (16)	
Self-assessment on weight	Very underweight	50.00% (51)	22.55% (23)	21.57% (22)	5.88% (6)	=0.009 *
	Slightly underweight	41.65% (227)	24.77% (135)	27.52% (150)	6.06% (33)	
	About right	38.04 (299)	25.06% (197)	31.42% (247)	5.47% (43)	
	Slightly overweight	27.18 (28)	36.89% (38)	31.07% (32)	4.85% (5)	
	Very overweight	26.67% (4)	26.67% (4)	46.67% (7)	0.00% (0)	
Desire to lose weight	No	34.30% (189)	25.95% (143)	33.39% (184)	6.35% (35)	=0.002 *
	Yes	41.96% (420)	25.47% (255)	27.37% (274)	5.19% (52)	
Dieting to lose weight	No	37.77% (494)	25.54% (334)	30.89% (404)	5.81% (76)	=0.002 *
	Yes	47.13% (115)	26.23% (64)	22.13% (54)	4.51% (11)	
Desire to increase physical activity	No	30.26% (82)	21.77% (59)	41.33% (112)	6.64% (18)	<0.001 **
	Yes	41.17% (527)	26.48% (339)	27.03% (346)	5.31% (68)	
Duration of sleep in 24 h period	<7 h	39.78% (144)	27.07% (98)	28.45% (103)	4.70% (17)	<0.001 **
	7–8 h	41.82% (419)	25.25% (253)	27.84% (279)	5.09% (51)	
	>8 h	25.14% (45)	24.02% (43)	40.78% (73)	10.06% (18)	

* Correlation is significant at the 0.05 level (2-tailed). ** Correlation is significant at the 0.01 level (2-tailed).

**Table 6:** Bivariate analysis for association between main concerns associated with the pandemic context and intention towards COVID-19 vaccination (*n* = 1552).

Main Concerns Associated with the Pandemic Context		COVID-19 Vaccination Status % (*n*)				*p* Value
		Got Vaccinated	Want to Get Vaccinated	Don’t Want to Get Vaccinated	Contraindication to Vaccine	
Fear of illness	No	37.14% (384)	22.92% (237)	34.53% (357)	5.42% (56)	<0.0001 **
	Yes	43.44% (225)	31.08% (161)	19.50% (101)	5.98% (31)	
Fear of death	No	38.49% (493)	24.59% (315)	31.69% (406)	5.23% (67)	=0.050 *
	Yes	42.80% (116)	30.63% (83)	19.19% (52)	7.38% (20)	
Fear of illness of close people	No	31.37% (181)	20.62% (119)	42.11% (243)	5.89% (34)	<0.0001 **
	Yes	43.90% (428)	28.62% (279)	22.05% (215)	5.44% (53)	
Fear of death of close people	No	33.99% (275)	21.38% (173)	38.32% (310)	6.30% (51)	<0.0001 **
	Yes	44.95% (334)	30.28% (225)	19.92% (148)	4.85% (36)	
Concerns regarding the capacity of the health system	No	35.97% (259)	23.89% (172)	35.00% (252)	5.14% (37)	=0.0003 *
	Yes	42.07% (350)	27.16% (226)	24.76% (206)	6.01% (50)	
Fear that the basic protective measures are not enough	No	38.80% (504)	24.71% (321)	31.25% (406)	5.23% (68)	=0.177
	Yes	41.50% (105)	30.43% (77)	20.55% (52)	7.51% (19)	
Concerns regarding the risk of becoming unemployed/ economic impact	No	41.58% (509)	24.75% (303)	28.27% (346)	5.39% (66)	=0.0001 **
	Yes	30.49% (100)	28.96% (95)	34.15% (112)	6.40% (21)	
Social isolation	No	39.54% (342)	26.01% (225)	29.02% (251)	5.43% (47)	=0.599
	Yes	38.86% (267)	25.18% (173)	30.13% (207)	5.82% (40)	
Social stigma associated with COVID-19 infection	No	40.13% (561)	24.75% (346)	29.90% (418)	5.22% (73)	=0.115
	Yes	31.17% (48)	33.77% (52)	25.97% (40)	9.09% (14)	
Family conflicts	No	39.67% (603)	25.46% (387)	29.34% (446)	5.53% (84)	=0.031 *
	Yes	18.75% (6)	34.38% (11)	37.50% (12)	9.38% (3)	

* Correlation is significant at the 0.05 level (2-tailed). ** Correlation is significant at the 0.01 level (2-tailed).

**Table 7:** Bivariate analysis for association between main information sources and intention towards COVID-19 vaccination (*n* = 1552).

Main Information Sources		COVID-19 Vaccination Status % (*n*)				Pearson’s *r*, *p* Value
		Got Vaccinated	Want to Get Vaccinated	Don’t Want to Get Vaccinated	Contraindication to Vaccine	
Traditional media	No	53.5% (326)	47.7% (190)	53.1% (243)	50.6% (44)	0.009, 0.714
	Yes	46.5% (283)	52.3% (208)	46.9% (215)	49.4% (43)	
The internet	No	40.9% (249)	44.0% (175)	37.3% (171)	44.8% (39)	0.013, 0.612
	Yes	59.1% (360)	56.0% (223)	62.7% (287)	55.2% (48)	
Friends, co-workers, family members	No	74.4% (453)	64.1% (255)	67.7% (310)	66.7% (58)	0.063 *, 0.013
	Yes	25.6% (156)	35.9% (143)	32.3% (148)	33.3% (29)	
Medical staff	No	28.2% (172)	42.5% (169)	57.9% (265)	29.9% (26)	−0.186 **, 0.000
	Yes	71.8% (437)	57.5% (229)	42.1% (193)	70.1% (61)	

* Correlation is significant at the 0.05 level (2-tailed). ** Correlation is significant at the 0.01 level (2-tailed).

**Table 8:** Bivariate analysis for association between COVID-19 symptoms and anxiety level (*n* = 313).

COVID-19 Symptoms	Correlations	Anxiety Index	Psychological Symptoms of Anxiety	Somatic Symptoms of Anxiety
Dyspnoea	Pearson correlation	0.205 **	0.127 *	0.220 **
	Sig. (2-tailed)	0.000	0.025	0.000
Fever	Pearson correlation	0.044	−0.008	0.065
	Sig. (2-tailed)	0.441	0.894	0.255
Headaches	Pearson correlation	0.146 **	0.120 *	0.144 *
	Sig. (2-tailed)	0.009	0.034	0.011
Muscle aches	Pearson correlation	0.126 *	0.108	0.121 *
	Sig. (2-tailed)	0.026	0.057	0.033
Sore throats	Pearson correlation	0.060	−0.006	0.084
	Sig. (2-tailed)	0.291	0.909	0.138
Loss of taste	Pearson correlation	0.016	0.041	0.005
	Sig. (2-tailed)	0.772	0.468	0.926
Loss of smell	Pearson correlation	−0.005	−0.001	−0.006
	Sig. (2-tailed)	0.930	0.992	0.921
Fatigue	Pearson correlation	0.165 **	0.148 **	0.160 **
	Sig. (2-tailed)	0.003	0.009	0.005

** Correlation is significant at the 0.01 level (2-tailed). * Correlation is significant at the 0.05 level (2-tailed). Abbreviations: Sig., significance level.

## Data Availability

The data presented in this study are available upon request from the corresponding author.

## Appendix A

**Table A1:** Values are depicted as percentages and numerical data.

Variables		Total (%) *n* = 1552
Gender	Female	1217 (78.4)
	Male	335 (21.6)
Residency	Urban	1197 (77.1)
	Peri-urban	118 (7.6)
	Rural	237 (15.3)
Marital status	Single	139 (9)
	Married	1049 (67.6)
	In relationship	288 (18.6)
	Widow	25 (1.6)
	Divorced	66 (4.3)
Household size and composition	Living alone	198 (12.8)
	Living with partner	1164 (75.0)
	Living with children	786 (50.6)
	Living with parents	187 (12.0)
	Living with siblings	57 (3.7)
	Others	50 (3.2)
Number of children	0	411 (26.5)
	1	645 (41.6)
	2	427 (27.5)
	≥3	69 (4.4)
Educational level	Secondary school	30 (1.9)
	Highschool	426 (27.4)
	University graduate	583 (37.6)
	Master graduate	411 (26.5)
	Doctoral degree	100 (6.4)
Mother’s educational level	No formal education	6 (0.4)
	Secondary school	312 (20.1)
	Highschool	816 (52.6)
	University degree	383 (24.7)
	Don’t know	34 (2.2)
Father’s educational level	No formal education	9 (0.6)
	Primary school	276 (17.8)
	Highschool	811 (52.3)
	University degree	407 (26.2)
	Don’t know	48 (3.1)
Occupational status	Unemployed	395 (25.4)
	Student	149 (9.6)
	Employed	766 (49.4)
	Freelance	113 (20.4)
	Retired	128 (8.2)
Revenues (with comparison to the pre-pandemic period)	Significantly decreased	87 (5.6)
	Decreased	322 (20.7)
	Stagnated	967 (62.3)
	Increased	151 (9.7)
	Significantly increased	25 (1.6)
Religious self-perception	Unreligious	539 (34.7)
	Religious	1013 (65.3)

**Table A2:** Values are depicted as percentages and numerical data. Comorbidities are not mutually exclusive. A total of 22 participants had two or more physical comorbidities.

Any Comorbid Physical Condition(s) %, (*n*)		
Yes	29.4	(456)
No	70.6	(1096)
**Physical condition(s), %, (*n*)**		
Endocrine and metabolic	7.8	(121)
Cardiovascular	7.1	(110)
Rheumatic and musculoskeletal	3.0	(46)
Hepatic	2.7	(42)
Neurological	1.6	(25)
Dermatological	1.5	(24)
Gynaecological	1.5	(23)
Pulmonary	1.2	(19)
Others	3.8	(59)
**Any comorbid mental condition(s) %, (*n*)**		
Yes	5.6	(87)
No	94.4	(1465)
**Mental condition(s), %, (*n*)**		
Major depression disorder	3.0	(47)
Anxiety disorder	1.9	(30)
Others	0.7	(10)

**Table A3:** Characteristics of the participants’ weight, physical activity, and sleep duration.

**I.** **Weight Status**					
Classification of BMI (kg/m^2^) According to WHO					
Category			BMI	%	*n*
Underweight (Severe thinness)			<16.0	0.3	(4)
Underweight (Moderate thinness)			16.0–16.9	1.4	(22)
Underweight (Mild thinness)			17.0–18.4	4.6	(72)
Normal range			18.5–24.9	52.8	(819)
Overweight (Pre-obese)			25.0–29.9	27.2	(422)
Obese (Class I)			30.0–34.9	10.2	(158)
Obese (Class II)			35.0–39.9	2.4	(37)
Obese (Class III)			≥40.0	1.2	(18)
**Self-assessment on weight, %, (*n*)**					
Very underweight				16	(1.0)
Slightly underweight				103	(6.6)
About right				786	(50.7)
Slightly overweight				545	(35.1)
Very overweight				102	(6.6)
**Desire to lose weight** **, %, (*n*)**					
Yes				64.5	(1001)
No				35.5	(551)
**Dieting to lose weight, %, (*n*)**					
Yes				15.7	(244)
No				84.3	(1308)
**II.** **Physical Activity**					
**Physical activity over the past 2 weeks?**			**What physical activity?**		
Yes No	% 58.6 41.4	*n* (909) (643)	a. Activity	%	*n*
			b. Walking	14.0	(218)
			c. Fitness	13.1	(204)
			d. Running/jogging	10.4	(161)
			e. Isometric exercises	7.9	(123)
			f. Cycling	3.5	(54)
			g. Others	3.7	(59)
**Desire to increase physical activity**					
Yes				82.5	(1280)
No				17.5	(271)
**III.** **V. Duration of sleep in 24-h period, %, *n***					
h. <7 h of sleep				23.4	(363)
i. 7–8 h of sleep				64.6	(1003)
j. >8 h of sleep				12.0	(186)

Note: BMI = Body Mass Index; WHO = World Health Organization. Mean BDI = 24.63, SD = 4.921. BMI is calculated by dividing weight (kg) by height (m^2^). The BMI categories for weight status are underweight = less than 18.5 kg/m^2^; normal weight = 18.5 to 24.9 kg/m^2^; overweight = 25 to 29.9 kg/m^2^; and obesity = greater than 30 kg/m^2^.

**Table A4:** Correlation between demographic characteristic and anxiety.

Demographic Variables		Anxiety Index (SAS)				
		Median	Mean	95.0% Lower CL for Mean	95.0% Upper CL for Mean	*p* Value
Gender	Female	43	45.21	44.6	45.81	
	Male	39	40.89	39.91	41.86	<0.001 **
Age groups	<25	46	48.33	46.42	50.24	
	25–34	43	44.66	43.88	45.44	
	35–44	40	42.78	41.75	43.82	
	45–54	41	43.73	41.69	45.78	
	55–64	41	42.87	41.28	44.45	
	>65	41	41.98	40.26	43.7	<0.001 **
Residency	Urban	41	43.94	43.34	44.53	
	Rural	41	44.85	42.78	46.92	
Educational level	Secondary school	55	54.33	49.6	59.07	
	Highschool	45	46.32	45.24	47.4	
	University graduate	41	43.65	42.81	44.49	
	Master graduate	40	42.43	41.51	43.35	
	Doctoral degree	42	43.91	41.93	45.89	=0.001 **
Occupational status	Employed	41	43.69	42.97	44.42	
	Student	46	47.34	45.38	49.29	
	Retired	41	42.55	41.05	44.05	
	Unemployment	50	49.97	47.14	52.8	
	Freelancer	39	42.21	40.09	44.33	=0.168
Being parents	No	44	45.72	44.59	46.86	
	Yes	41	43.76	43.17	44.34	=0.007 **
Number of children	0	44	45.73	44.6	46.86	
	1	41	43.75	42.96	44.54	
	2	41	43.99	43.02	44.96	
	≥3	40	42.92	40.25	45.6	=0.016 *
Revenues	Significantly decreased	48	48.99	46.16	51.82	
	Decreased	45	46.87	45.57	48.17	
	Stagnated	41	43.22	42.61	43.83	
	Increased	41	42.89	41.34	44.45	
	Significantly increased	38	43.44	38.51	48.37	<0.001 **
Family background	Wealthy	37	40.50	35.71	45.29	<0.001 **
	Quite well off	40	42.38	41.68	43.07	
	Not very well off	44	46.06	45.28	46.84	
	Quite poor	48	46.93	41.45	52.42	
Satisfied with their own lives	Extremely satisfied	38	38.64	37.76	39.52	
	Moderately satisfied	41	44.03	43.43	44.62	
	Neither satisfied nor dissatisfied	50	51.31	49.68	52.94	
	Moderately dissatisfied	56	56.67	53.13	60.22	
	Extremely dissatisfied	61	61.82	51.36	72.28	<0.001 **

Note. SAS = Zung Self-Rating Anxiety Scale; CL = Confidence Level. Anxiety index range from 0 to 100 and the threshold values: absence of anxiety <45, mild intensity anxiety 45–59, moderate intensity anxiety 59–74 and severe intensity anxiety >75. * Correlation is significant at the 0.05 level (2-tailed). ** Correlation is significant at the 0.01 level (2-tailed).
